# Microelectrode recordings from the human cervical vagus nerve during maximal breath‐holds

**DOI:** 10.1113/EP092890

**Published:** 2025-06-11

**Authors:** Vaughan G. Macefield, Anthony R. Bain, Matthew I. Badour, Marko Kumric, Ivan Drvis, Otto F. Barak, Josko Bozic, Zeljko Dujic

**Affiliations:** ^1^ Department of Neuroscience Monash University Melbourne Victoria Australia; ^2^ Baker Heart and Diabetes Institute Melbourne Victoria Australia; ^3^ Faculty of Human Kinetics University of Windsor Windsor Ontario Canada; ^4^ Department of Kinesiology McMaster University Hamilton Ontario Canada; ^5^ Department of Pathophysiology University of Split School of Medicine Split Croatia; ^6^ Faculty of Kinesiology University of Zagreb Zagreb Croatia; ^7^ Department of Physiology, Faculty of Medicine University of Novi Sad Novi Sad Serbia; ^8^ Department of Integrative Physiology University of Split School of Medicine Split Croatia

**Keywords:** apnoea, asphyxia, breath‐hold, microneurography, vagus nerve

## Abstract

Voluntary breath‐holds can be sustained for a long time following training, but ultimately, regardless of duration, the asphyxic break‐point is reached and the apnoea terminated. The physiological changes occurring during the apnoea include a marked increase in sympathetically‐mediated vasoconstriction in non‐essential organs, such as skeletal muscle, spleen and kidney, while the brain is protected by a marked increase in perfusion. What is not understood is what happens to cardiac vagal activity. Here, we performed microelectrode recordings from the right cervical vagus nerve in healthy participants [both trained breath‐hold divers (*n* = 10) and untrained controls (*n* = 10)] during tidal breathing, slow‐deep breathing, an inspiratory‐capacity apnoea and an end‐expiratory apnoea. Using cross‐correlation analysis of multi‐unit neural activity, we tested the hypothesis that breath‐hold divers would have greater cardiac modulation of vagal activity, which primarily reflects the discharge of cardiac afferents, particularly during a maximal apnoea. We showed that there were no differences in cardiac modulation of vagus nerve activity either during tidal breathing or during any of the respiratory manoeuvres, nor was there a difference in cardiac modulation during the static phase of a maximal apnoea or when involuntary breathing movements occurred before reaching the asphyxic break‐point. We conclude that changes in vagal sensory inputs from the heart are not responsible for the marked tolerance to asphyxia shown by breath‐hold divers.

## Abstract (highlights)

- **What is the central question of this study?**
  Severe asphyxia leads to purposeful increases in sympathetic vasoconstrictor drive to reduce blood flow to non‐exercising muscle and other non‐essential organs, but it is not known how the parasympathetic nervous system responds during severe asphyxia.
- **What is the main finding and its importance?**
  Microelectrode recordings from the right vagus nerve in humans revealed that elite breath‐hold divers did not have greater cardiac and respiratory modulation of vagal activity than control participants, arguing against adaptations in the vagus nerve of breath‐hold divers.

## 1 INTRODUCTION

The capacity of the body to tolerate asphyxia is remarkable. Increases in CO_2_ and decreases in O_2_ generate purposeful physiological responses that aim to preserve the delivery of O_2_ to and removal of CO_2_ from the organs that need it most, i.e., the brain and heart. As such, blood flow to non‐essential organs, such as the gut, skin, kidneys and skeletal muscle, is reduced. Progressive asphyxia, induced by a maximal breath‐hold performed at the end of a normal expiration, causes muscle sympathetic nerve activity (MSNA; a direct measure of vasoconstrictor drive to skeletal muscle) to increase progressively up to the asphyxic breaking point, at which point the MSNA is inhibited as soon as the subject takes a breath (Hardy et al., 1994; Leuenberger et al., 1995; Muenter Swift et al., 2003; Seitz et al., 2013; Watenpaugh et al., 1999). During the apnoea, one can assess the influence of increasing chemoreceptor activity on MSNA in the absence of ongoing ventilation (van de Borne et al., 2001), and the immediate suppression of MSNA during the first breath does not depend on suppression of chemical drive (Seitz et al., 2013). It is well known that breath‐holding time is determined by the increase in CO_2_, rather than the decrease in O_2_, and by lung volume (Godfrey et al., 1969; Godfrey and Campbell, 1969; Kelman and Wann, 1971). Moreover, participants can continue to hold their breath after a single breath of pure N_2_, during which MSNA continues to increase (Seitz et al., 2013). It is also known that lung inflation also inhibits MSNA (Seals et al., 1990; Macefield & Wallin, 1995a, 1995b; St. Croix et al., 1999; van de Borne et al., 2001) and that high‐volume ventilation attenuates the increases in MSNA produced by hypoxia and/or hypercapnia (Somers et al., 1989a, 1989b). Nevertheless, with prolonged apnoeas [especially those performed by elite freedivers, also known as breath‐hold divers (BHDs)], a series of involuntary breathing movements (IBMs) occur against the closed glottis before the larynx opens and the first breath is taken. It is well established that the progressive increase in sympathetic vasoconstrictor drive during an end‐expiratory apnoea is a reflex caused by progressive hypoxia and hypercapnia, as detected by the peripheral and central chemoreceptors (Breskovic, Ivancev et al., 2010; Dujic et al., 2008; Halliwell & Minson, 2002; Halliwell et al., 2003; Heusser et al., 2009; Marshall, 1994; Saito et al., 1988; Somers et al., 1989a, 1989b; Steinback, Breskovic et al., 2010a; Steinback, Salmanoiur et al., 2010b).

Although marine mammals such as seals, dolphins and whales, routinely hold their breath during dives, the capacity of humans, who do not have the rich muscle myoglobin stores of marine mammals (Berenbrink et al. 2021), to sustain voluntary apnoeas is remarkable, with divers able to hold their breath for >10 min (for review, see Bain, Drvis et al., 2018); the present world record for a maximal static apnoea (i.e., voluntary breath‐hold without active diving) is 11 min 54 s without or 24 min 36 s with pre‐breathing of O_2_ (www.aidainternational.org/freediving). Researchers in our laboratory have been investigating the physiological adaptations of BHD for the last 15 years, and we could not find any differences in resting blood pressure, heart rate, haemoglobin oxygen saturation, MSNA or vascular resistance between BHDs and untrained control subjects (Heusser et al., 2009), nor could we demonstrate differences in peripheral chemosensitivity in elite apnoea divers before (Breskovic, Ivancev et al., 2010) or during the training season (Breskovic, Valic et al., 2010) or differences in cardiac structure and cardiopulmonary function between divers and control subjects at baseline (Kelly et al., 2022). Moreover, there were no differences between the two groups with respect to central chemoreflex sensitivity (Dujic et al., 2008) or cerebrovascular reactivity to hypercapnia (Ivancev et al., 2007). In the divers, cerebral blood flow and O_2_ delivery increased progressively during a maximal apnoea right up to the asphyxic break‐point, when arterial oxygen saturation had fallen to 40%–50% (Willie et al., 2015). Moreover, permeability of the blood–brain barrier is not compromised in the divers (Bain, Ainslie et al., 2018). Finally, direct microelectrode recordings of sympathetic outflow to the muscle vascular bed revealed no differences in MSNA between the two groups at rest (Dujic et al., 2008).

Nevertheless, in all of these and other studies, significant changes occurred at the break‐point of a maximal static apnoea in BHDs. For example, MSNA was increased 700%–800% from baseline (Heusser et al., 2009), with recruitment of large postganglionic muscle vasoconstrictor neurones (Steinback, Breskovic et al., 2010a; Steinback, Salmanoiur et al., 2010b) and sympathetically‐mediated constriction of the spleen; indeed, splenectomized individuals are not able to hold their breath as long as intact control participants (Bakovic et al., 2003). We do know that the mammalian diving reflex (comprising sympathetically‐mediated constriction of systemic resistance vessels and parasympathetically‐mediated bradycardia) promotes oxygen conservation to maintain cerebral and cardiac function, but we also know from measurements of heart rate variability (HRV) that it is likely that there is an increase in sympathetic drive to the heart during active diving (Kiviniemi et al., 2012) that counteracts any vagally‐mediated decrease in heart rate and increase in HRV seen during static dives, in which a maximal apnoea is performed in the absence of exercise and without facial cooling to evoke the diving reflex (Kiviniemi et al., 2012; Lemaître et al., 2008). What we do not know is what sensory information arises from the heart during a maximal breath hold and how this might contribute to the control of the cardiovascular system that ultimately allows tolerance to marked reductions in O_2_ saturation. Here, we undertook an investigation into the physiology of the human vagus nerve during maximal breath‐holds, using direct microelectrode recordings of neural traffic via a tungsten microelectrode inserted percutaneously into the cervical vagus nerve (Farmer et al., 2025; Ottaviani et al., 2020; Patros et al., 2024, 2022). We used cross‐correlation analysis of multi‐unit neural activity, as described previously (Patros et al., 2022), to test the primary hypothesis that elite divers would have greater cardiac modulation of vagal activity.

## 2 MATERIALS AND METHODS

**FIGURE 1:**
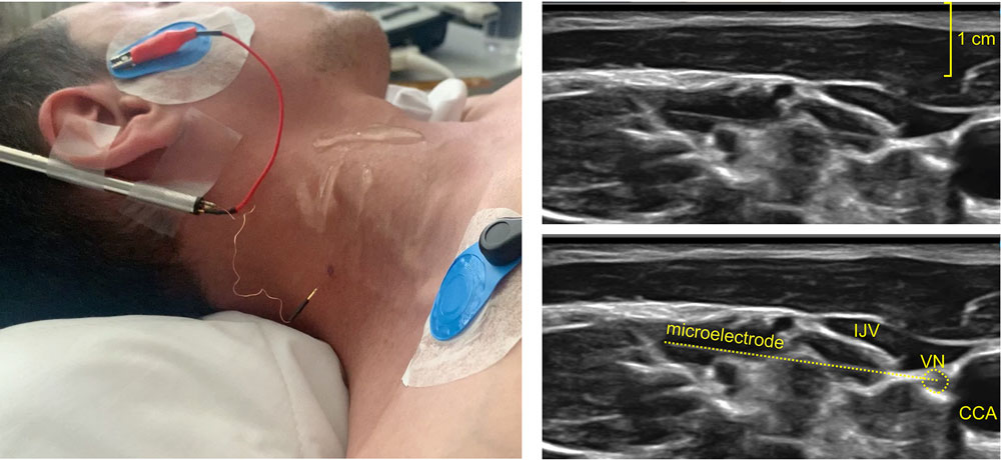
Microelectrode inserted into the right vagus nerve in a 26‐year‐old male breath‐hold diver (BHD 2) performing a maximal inspiratory apnoea (note the distended external jugular vein). Ultrasound gel can be seen on the neck. The ultrasound images at the right show the raw and annotated images of the microelectrode, with the tip in situ within the VN. The nerve is lateral to the CCA and posterior to the IJV. Abbreviations: CCA, common carotid artery; IJV, internal jugular vein; VN, vagus nerve.

Studies were performed on 10 male elite apnoea divers (26–47 years old) and 9 male and 2 female control participants (21–27 years old). All participants provided informed written consent to the procedures, which were conducted under the approval of the University of Split School of Medicine Ethics committee (Class: 003‐08/21‐03/0003; Reg. no.: 2181‐198‐03‐04‐21‐0017) and conformed to the *Declaration of Helsinki*, with the exception that patients were not registered in a database. Some of the present participants were referred to in a recent study in which we examined the risk of bradycardia and asystole following microelectrode penetration of the vagus nerve, but that study did not present a detailed analysis of the neural data (Patros et al., 2024). Details of our approach to recording from the vagus nerve have been described previously (Ottaviani et al., 2020; Patros et al., 2024, 2022), with all the present recordings being performed on the right vagus nerve. Briefly, with participants lying supine and the neck rotated comfortably to the left, a 12 MHz linear ultrasound probe (T3200, Terason, Burlington, MA, USA) was used to the measure the intima–media thickness of the carotid artery posterior wall, ∼2 cm caudal to the bifurcation and to assess whether there were any evidence of atherosclerosis or plaque, not proceeding in cases if the intima–media thickness was >1 mm. The vagus nerve, usually located dorsolateral to the common carotid artery and posterior to the internal jugular vein, was then imaged, and an optimal insertion site (defined as a site in which a clear linear path to the vagus nerve could be achieved) was chosen caudal to the carotid bifurcation. A sterile tungsten microelectrode (Frederick Haer, Bowdoin, ME, USA; diameter, 200 µm; length, 40–50 mm) was inserted into the skin at the posterior border of the sternocleidomastoid muscle and manually directed to the vagus nerve under ultrasound guidance, ensuring a clear trajectory that avoided the posterior wall of the internal jugular vein (Figure 1). An uninsulated reference electrode was inserted subdermally ∼1–2 cm from the recording microelectrode.

Neural activity was amplified (20 000×, 0.3–5 kHz) using a low‐noise amplifier (NeuroAmpEX, ADInstruments, Sydney, NSW, Australia) and sampled at 20 kHz via a computer‐based data acquisition and analysis system (PowerLab 16/35; LabChart 8 software, ADInstruments). After penetration of the nerve, occasionally heralded by the generation of ectopic impulses (insertion discharges; Patros et al., 2024) and confirmed by ultrasound imaging of the tip of the microelectrode within the nerve, the ultrasound probe was withdrawn and any spontaneous neural activity recorded. If the fascicle of the nerve was silent, the position of the microelectrode tip was adjusted manually until activity with cardiac‐ and/or respiratory‐related activity was found, as described previously (Patros et al., 2022). The ECG was recorded using Ag/AgCl surface electrodes on the chest and sampled at 2 kHz (BioAmplifer; ADInstruments), blood pressure was recorded continuously via finger photoplethysmography (Finometer Pro, Finapres Medical Systems, The Netherlands) and intermittently via sphymomanometry and sampled at 400 Hz, and respiration was sampled at 100 Hz using a strain‐gauge transducer wrapped around the chest (Pneumotrace; UFI, Morro Bay, CA, USA). Oxygen saturation was measured using a pulse oximeter attached to a finger.

For each intrafascicular recording site, the following manoeuvres were performed: (1) baseline activity with tidal breathing (5 min); (2) slow‐deep breathing at 5 breaths/min (2 min); (3) an inspiratory‐capacity apnoea, in which the lungs were held maximally inflated against a closed glottis for 40 s; and (4) an end‐expiratory apnoea, in which participants held their breath at functional residual capacity for 40 s. In addition, the BHDs performed two to three practice breath‐holds before attempting a maximal apnoea of varying duration, preceding this with a period of hyperventilation that excluded glossopharyngeal insufflation ‘lung packing’, because this would cause large neck movements and dislodge the microelectrode. In order to improve their capacity to hold their breath via accentuating the mammalian dive response, a gel ice‐pack was placed over their face immediately prior to the maximal attempted apnoea and released after the break‐point had been reached.

Action potentials recorded from the vagus nerve were extracted using window discriminator software (Spike Histogram, ADInstruments), which separated the narrow axonal spikes (with a half‐width of 0.2–0.5 ms) from any broad motor‐unit potentials from contracting neck muscles. The same software was used to detect the R‐waves of the ECG, while the Cyclic Measurements extension was used to detect the inspiratory peaks of respiration. Standard pulses from these timing events were used to construct auto‐correlation histograms (correlograms) of the ECG and inspiratory peaks, and cross‐correlation histograms were constructed between the neural spikes and the cardiac and respiratory timing events; 50 ms bins were used for all signals. The histogram data were exported as text to a statistical and graphical analysis program (Prism v.10 for Mac, GraphPad Software, USA), to fit the data to a mathematical function (a smoothed polynomial). Quantification of cardiac and respiratory modulation was performed by measuring the difference in the number of spikes on the smoothed curve at the peak of the modulation closest to time 0 and at the trough (to the right of the peak). These were then expressed as a percentage by using the following formula: modulation index (%) = [(peak − trough)/peak] × 100. All statistical analyses were performed using Prism v.10 software.

## 3 RESULTS

Successful intraneural recordings were made from the right vagus nerve in all 10 BHDs and 10 of 11 control participants. Demographics are provided in Table 1. Although the divers were older than the control subjects (the latter were a convenience sample of university students), all participants were healthy and fit. One 41‐year‐old diver (BHD 5) had untreated high blood pressure but was otherwise healthy and not limited in performing the manoeuvres. All participants tolerated the procedure well, with none reporting significant discomfort other than a dull ache as the needle passed through fascial planes and muscle. In the one control participant in whom we did not manage to record from the vagus nerve, the microelectrode was inserted low in the neck and penetrated the superior trunk of the brachial plexus, as evidenced by the participant reporting radiating paraesthesiae into the index finger; this experiment was discontinued.

**TABLE 1:** Participant demographics.

Participant	Sex	Age (years)	Height (cm)	Weight (kg)	BMI (kg/m^2^)	HR (beats/min)	Systolic BP (mmHg)	Diastolic BP (mmHg)
CTL 1	M	21	186	75	21.6	56	122	88
CTL 2	M	21	173	67	25.2	69	124	76
CTL 3	M	23	181	89	24.7	62	126	84
CTL 4	M	23	180	70	25.6	47	122	80
CTL 5	M	19	190	91	21.7	54	132	84
CTL 6	M	25	193	93	25.4	47	128	88
CTL 7	F	25	168	59	25.3	63	114	78
CTL 8	M	21	171	60	25.0	64	122	80
CTL 9	F	21	171	64	21.9	79	122	80
CTL 10	M	27	189	86	22.8	74	134	88
Mean ± SD		22.6 ± 2.5	180.2 ± 9.1	75.4 ± 13.3	23.9 ± 1.7	61.6 ± 10.8	124.6 ± 5.7	82.6 ± 4.4
								
BHD 1	M	41	178	76	22.5	57	134	84
BHD 2	M	26	175	62	22.8	62	110	80
BHD 3	M	47	192	95	24.9	56	120	78
BHD 4	M	42	193	89	23.9	60	119	59
BHD 5	M	41	180	80	23.1	60	135	90
BHD 6	M	28	180	83	22.8	53	116	78
BHD 7	M	39	187	92	25.4	64	116	82
BHD 8	M	36	173	73	21.5	51	132	84
BHD 9	M	30	177	76	22.8	87	132	88
BHD 10	M	34	189	71	23.0	58	124	78
Mean ± SD		36.4 ± 6.8 ^****^	181.4 ± 6.9	79.7 ± 10.2	23.3 ± 1.2	60.5 ± 10.0	123.8 ± 8.9	80.1 ± 8.5

*Note*: Blood pressure measurements were obtained via sphygmomanometry.

Abbreviations: BHD, breath‐hold diver; BMI, body mass index; BP, blood pressure; CTL, control; HR, heart rate.

**** Significant difference between CTL and BHD (*p* < 0.0001).

As described previously (Patros et al., 2022), not all sites within the right vagus nerve had ongoing activity that was modulated by the cardiac rhythm and/or respiration, but we could usually encounter such sites by adjusting the microelectrode location. Given that respiratory‐related activity (both afferent and efferent) is prevalent in the vagus nerve, we were primarily interested in finding intrafascicular sites displaying cardiac rhythmicity, because this reflects parasympathetic neural activity going to (efferent) or coming from (afferent) the heart.

**FIGURE 2:**
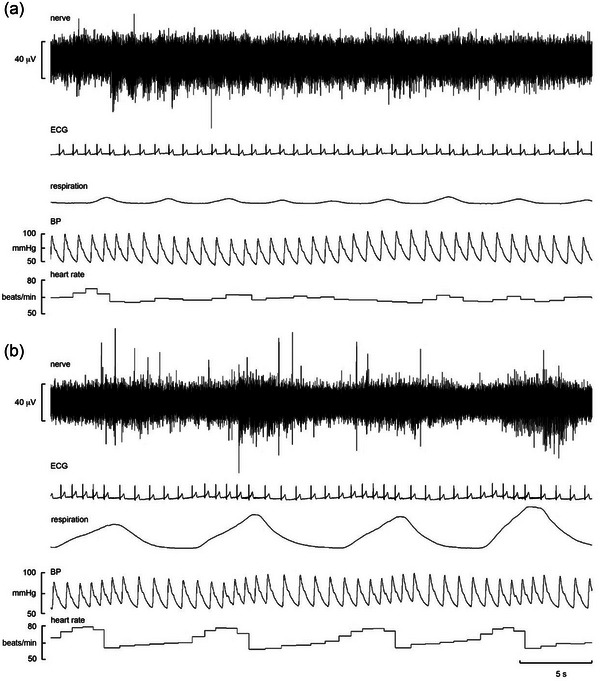
Microelectrode recordings of vagal activity (top trace), ECG, respiration, blood pressure and heart rate from a 26‐year‐old male breath‐hold diver (BHD 2) at rest (a) and during slow‐deep breathing (b). Note the clear cardiac‐locked bursts of vagal activity, dominated by negative‐going spikes, at rest; these were augmented during slow‐deep breathing, which constrained the bursts to occurring primarily within the inspiratory phase. Abbreviation: BP, blood pressure.

Experimental records from an intrafascicular site during quiet breathing in a 26‐year‐old male BHD are shown in Figure 2. It can be seen that multi‐unit bursts of negative‐going spikes occur with clear cardiac rhythmicity at rest (Figure 2a) and that during slow‐deep breathing, the cardiac modulation was augmented during inspiration and largely constrained to occur within the inspiratory phase of the respiratory cycle (Figure 2b). The fact that the spikes composing these bursts were negative‐going means that they were generated by unmyelinated axons (Macefield, 1998b; Macefield et al., 1994); the slow‐conducting nature of these axons might contribute to the lag from the start of inspiration but is more likely to reflect the events leading to the bursts. The reduction in intrathoracic pressure during inspiration will lead to an increase in venous return, supporting our interpretation that these bursts originated in atrial stretch receptors responding to the increase in atrial volume (i.e., low‐pressure baroreceptors). As expected, these would fire physically with atrial distension during diastole, and this would be augmented during inspiration.

**FIGURE 3:**
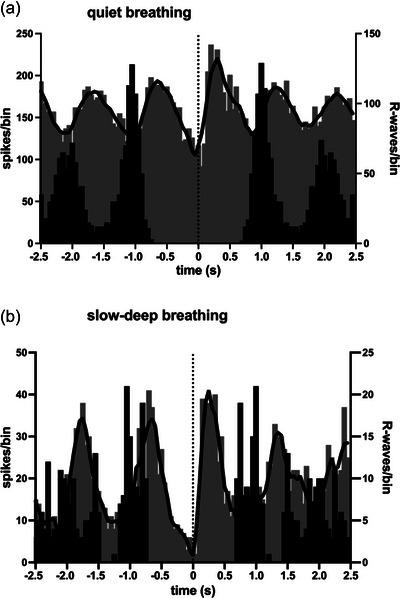
Cross‐correlation histograms (grey bars) between vagal activity and the ECG in a fascicle of the right vagus nerve, together with autocorrelation histograms of the ECG (black bars) from a 26‐year‐old male breath‐hold diver (BHD 2; same as in Figure 1). Cardiac modulation of vagal activity at rest (a) was augmented during slow‐deep breathing (b). Bin width = 50 ms for both sets of histograms. Time 0 corresponds to the triggering R‐wave of the ECG. The continuous black lines are smoothed polynomials fitted to the cross‐correlation histogram to illustrate better the cardiac rhythmicity of the vagal activity. The recording period during slow‐deep breathing was shorter than during tidal breathing, hence the lower number of spikes and the difference in the *y*‐axes.

Cardiac modulation of vagal activity at rest and during slow‐deep breathing was quantified by constructing cross‐correlation histograms (correlograms) between the extracted neural spikes and the R‐waves of the ECG. Cross‐correlograms for the bursts of negative‐going spikes recorded in the BHD shown in Figure 2 are illustrated in Figure 3, together with the autocorrelograms for the ECG to define the cardiac rhythmicity.

Modulation indices were calculated for 27 vagal sites in 10 healthy control subjects and 10 sites in 10 BHDs during quiet breathing. It should be noted that fewer intrafascicular sites were obtained in the divers, not because of technical difficulties, but because the BHDs (once we had obtained a suitable intrafascicular site) wanted to practice breath‐holding for the sake of the study; we did not ask the healthy participants to attempt maximal apnoeas. This afforded us more time to explore fascicles of the vagus nerve in the control participants and also provided data with which we could compare the BHDs with the healthy control subjects.

The magnitude of cardiac modulation varied markedly across intrafascicular sites, both in the control participants (1.8%–88.3%) and in the BHDs (1.7%–56.7%); the high variability across participants extended to a high variability across sites within the same participant, at least as assessed in the control participants (there were more sites sampled in this cohort). Mean modulation indices are shown in Table 2. On average, during tidal breathing, cardiac modulation was ∼21% for both the controls and the divers; nor were there significant differences in the magnitude of cardiac modulation between the two groups during 2 min of slow‐deep breathing, but (as noted above) the variability across sites was high.

**TABLE 2:** Mean ± SD modulation indices (percentage, [range]) for cardiac and respiratory modulation of vagal activity (see Materials and Methods) recorded in the 10 controls (CTLs) and 10 breath‐hold divers (BHDs).

Manoeuvre	CTL	BHD	P
Cardiac modulation of vagal activity			
Tidal breathing	21.3 ± 21.9 [1.8–83.3] (*n* = 27)	20.6 ± 18.3 [1.7–56.7] (*n* = 9)	0.9428 (MW)
Slow‐deep breathing	14.8 ± 10.5 [3.9–32.4] (*n* = 11)	34.7 ± 37.1 [4.0–100.0] (*n* = 9)	0.2947 (MW)
Inspiratory‐capacity apnoea	26.8 ± 15.3 [5.9–66.7] (*n* = 21)	40.7 ± 31.8 [14.3–100.0] (*n* = 10)	0.1092 (Ut)
End‐expiratory apnoea	20.7 ± 4.2 [14.3–26.3] (*n* = 6)	35.9 ± 29.9 [14.5–95.0] (*n* = 6)	0.2468 (Ut)
Maximal apnoea (static)		22.5 ± 19.7 [3.9–63.6] (*n* = 12)	
Maximal apnoea (IBM)		26.4 ± 29.0 [6.1–100.0] (*n* = 10)	
**Respiratory modulation of vagal activity**			
Tidal breathing	35.2 ± 14.1 [17.2–60.0] (*n* = 9)	52.7 ± 23.5 [28.6–94.4] (*n* = 10)	0.0931 (Pt)
Slow‐deep breathing	55.8 ± 14.7 [34.4–78.3] (*n* = 10)	65.2 ± 25.1 [28.1–100.0] (*n* = 10)	0.3215 (Pt)

Abbreviations: IBM, involuntary breathing movements; MW, Mann–Whitney *U* test; Pt, Student's paired *t*‐test; *n*, number of intraneural sites analysed; Ut, Student's paired *t*‐test.

Respiratory modulation of vagal activity was quantified during tidal breathing and 2 min of slow‐deep breathing. The mean respiratory modulation index at rest was significantly higher than the mean cardiac modulation index, both for the control participants (35.2% ± 14.1% vs. 21.3% ± 21.9%; *p* = 0.0098, Mann–Whitney *U*‐test) and for the divers (52.7% ± 23.5% vs. 20.6% ± 18.3%; *p* = 0.0043, Mann–Whitney *U*‐test), but there was no difference in the magnitude of respiratory modulation between the two groups (*p* = 0.0931, Student's unpaired *t*‐test). As expected, respiratory modulation of vagal activity increased significantly during slow‐deep breathing for the controls (*p* = 0.004, Student's paired *t*‐test) but, surprisingly, not for the BHDs (*p* = 0.1891, Student's paired *t*‐test), because their respiratory modulation was already high. Nevertheless, there was no difference in the magnitude of respiratory modulation between the control subjects and BHDs (Table 2).

To exclude the phasic effects of respiration, cardiac rhythmicity of vagal activity was also measured during a 40 s inspiratory‐capacity apnoea, in which the lungs were held maximally inflated against a closed glottis, and a 40 s end‐expiratory apnoea, in which participants held their breath at functional residual capacity. On average, as with baseline activity and slow‐deep breathing, the mean modulation indices during the inspiratory‐capacity apnoea were not significantly different between the control participants and the BHDs, although the variability across sites was high; the same was true for the end‐expiratory apnoea (Table 2). Moreover, there were no differences in the magnitude of cardiac modulation between the inspiratory‐capacity apnoea and end‐expiratory apnoea in either the controls (*p* = 0.3487; Student's unpaired *t*‐test) or the BHDs (*p* = 0.7711; Student's unpaired *t*‐test). This tells us that the cardiac modulation of vagal activity was independent of phasic respiratory influences and was not significantly different between the controls and the divers.

**FIGURE 4:**
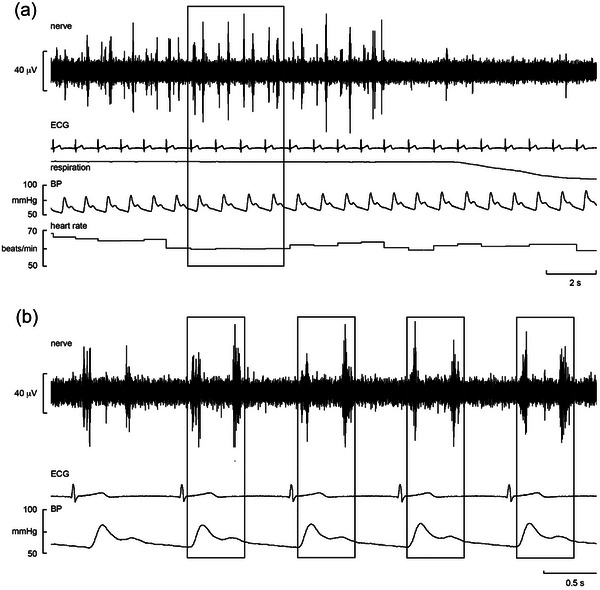
(a) Microelectrode recordings of vagal activity (top trace), ECG, respiration, blood pressure and heart rate from a 26‐year‐old male breath‐hold diver (BHD 2) during the final stage of an inspiratory‐capacity apnoea. (b) Expanded section from (a), with the blood pressure trace shifted back in time by 150 ms to illustrate the temporal relationships between the neural bursts and systole and the dichrotic notch. Abbreviation: BP, blood pressure.

Nevertheless, as indicated by the numerical differences in modulation indices between the two groups, we did encounter vagal sites in which cardiac modulation did appear to be higher in the BHDs. One such example, shown during the final stages of an inspiratory‐capacity apnoea, is shown in Figure 4. This intrafascicular site, obtained from the same diver illustrated in Figures 2 and 3, was interesting because there were two bursts of neural activity per cardiac cycle. It should be noted that blood pressure was very low in this individual, because he had just recovered from an apparent psychogenic vasovagal episode (described by Patros et al., 2024) but was happy to continue with the recording. Given that continuous blood pressure was recorded non‐invasively from a finger and that it takes ∼0.15 s for the arterial pressure wave to travel from the left ventricle to the finger, in Figure 4b the blood pressure has been shifted 150 ms back in time such that the neural bursts align with the blood pressure recording (as a proxy marker of the increases in ventricular pressure occurring within the left ventricle); it can be seen that the first burst is associated with systole and the second with the dicrotic notch in the blood pressure waveform.

**FIGURE 5:**
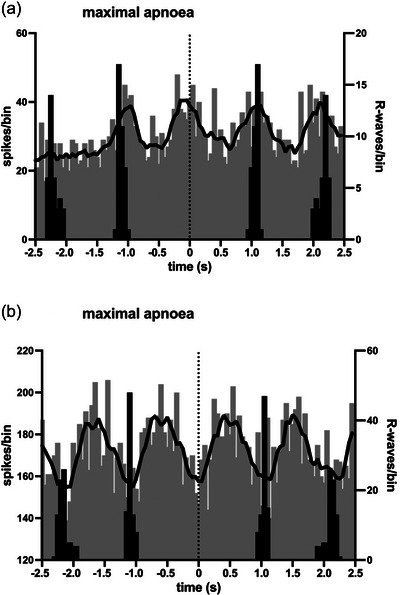
Cross‐correlation histograms (grey bars) between two intrafascicular sites of the right vagus nerve and the ECG, together with autocorrelation histograms of the ECG (black bars), recorded from a 34‐year‐old male breath‐hold diver (BHD 10). Note that the primary modulation of vagal activity in (a) occurred immediately before the R‐wave, whereas the peak modulation in (b) occurred in the middle of the cardiac cycle. Bin width = 50 ms for both sets of histograms. Time 0 corresponds to the triggering R‐wave of the ECG. The continuous black lines are the smoothed polynomials fitted to the cross‐correlation histograms to illustrate better the cardiac rhythmicity of the vagal activity.

All BHDs were asked to hold their breath for as long as possible, which, as they had trained to do, was preceded by a brief period of hyperventilation to maximize pulmonary O_2_ capacity and blow off their CO_2_. Given that most control participants could hold their breath for <1 min, we did not ask them to perform a maximal apnoea. Figure 5 shows two cross‐correlation histograms from two sites recorded from a BHD performing a maximal apnoea. It can be seen that although both sites exhibited cardiac rhythmicity, the peak modulation occurred immediately prior to the R‐wave in Figure 5a, whereas symmetrical peaks occurred on either side of the R‐wave in Figure 5b, no doubt reflecting the different populations of axons composing each recording site.

**FIGURE 6:**
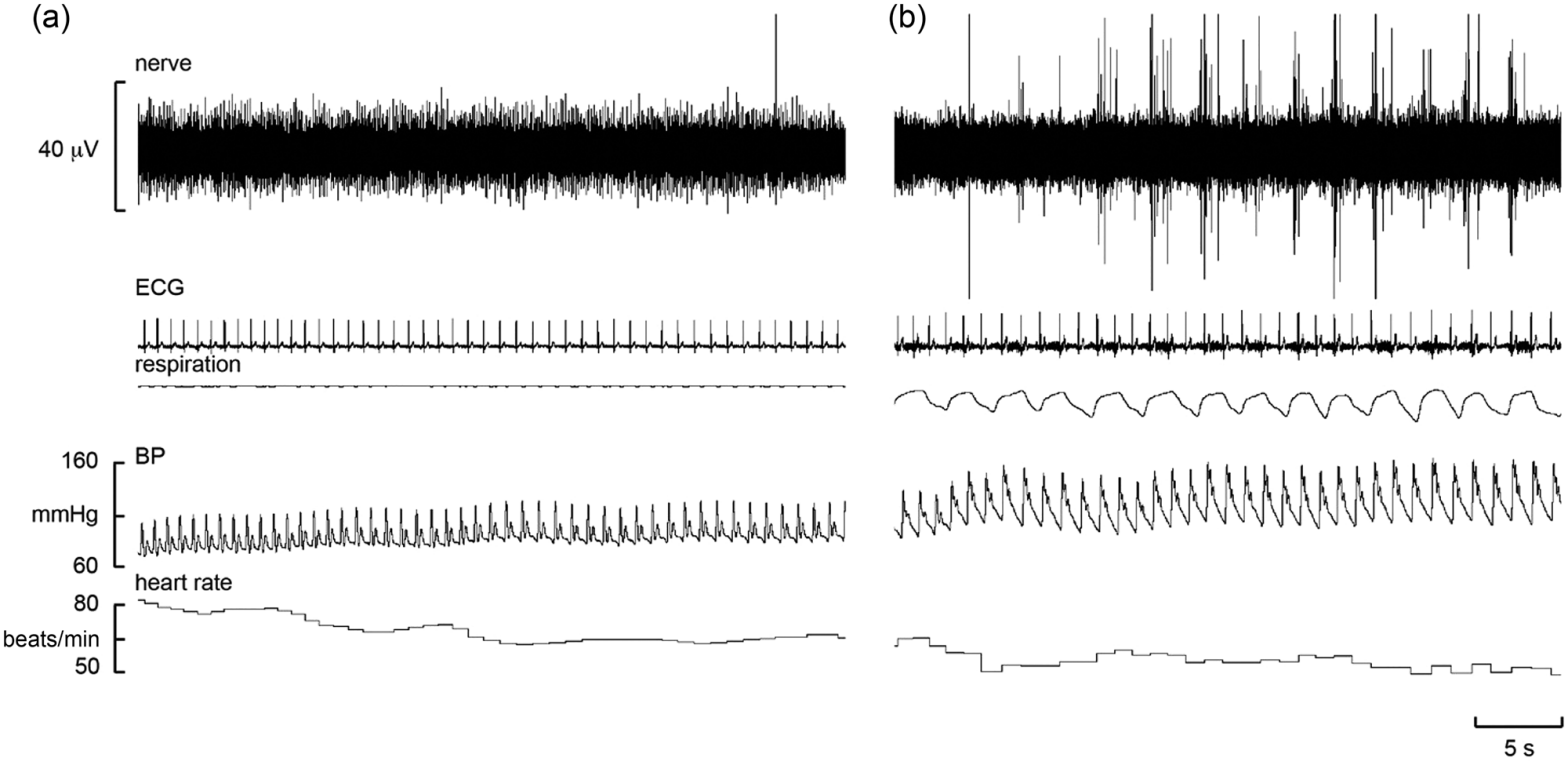
Microelectrode recordings of vagal activity (top trace), ECG, respiration, blood pressure and heart rate from a 47‐year‐old male breath‐hold diver (BHD 3) performing a maximal apnoea. (a) The traces commence 10 s after the start of the apnoea. (b) The traces commence 3 min 20 s into the apnoea. Abbreviation: BP, blood pressure.

**FIGURE 7:**
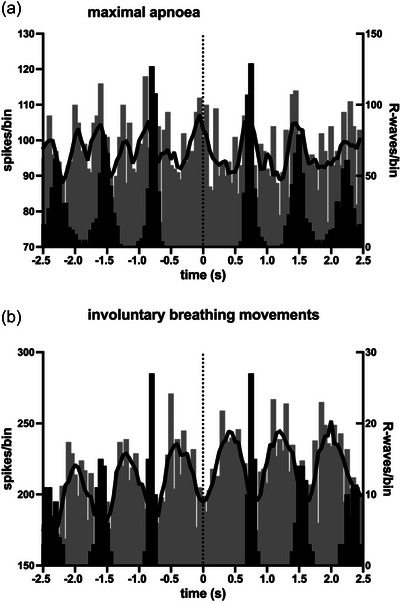
Cross‐correlation histograms (grey bars) between vagal activity and the ECG in a fascicle of the right vagus nerve, together with autocorrelation histograms of the ECG (black bars) from a 26‐year‐old male breath‐hold diver (BHD 2) during the static phase of a maximal apnoea (a) and during the later stage, in which involuntary breathing movements occurred (b). Bin width = 50 ms for both sets of histograms. Time 0 corresponds to the triggering R‐wave of the ECG. The continuous black lines are the smoothed polynomials fitted to the cross‐correlation histograms to illustrate better the cardiac rhythmicity of the vagal activity.

Experimental records from a BHD performing a maximal breath‐hold are shown in Figure 6. It can be seen that, before reaching the asphyxic break‐point, IBMs occurred; because these efforts were made against a closed glottis, they did not satisfy the chemical urge to breathe but nevertheless indicate that the participant was struggling to sustain the apnoea. Cross‐correlograms from this BHD, constructed during the static phase of the apnoea prior to the period of IBMs and during the IBMs, are shown in Figure 7a, b, respectively. Cardiac modulation was more marked during the IBMs, although it is also apparent from the autocorrelograms that heart rate was more stable during the IBMs in this participant. This BHD could sustain an apnoea for 7 min 14 s in the present laboratory environment. Individual and mean parameters of the apnoeas are shown in Table 3. Mean heart rates during the later part of the apnoea (during the phase in which IBMs occur) were lower than during the earlier (static) phase; mean blood pressure was also higher during this phase than during the static phase. Peak O_2_ desaturation at the end of the apnoea ranged from 48% to 91% (69.6% ± 18.5%).

**TABLE 3:** Characteristics of maximal apnoeas performed by the breath‐hold divers.

Participant	IBM onset (s)	IBM duration (s)	Apnoea duration (s)	HR static (beats/min)	HR IBM (beats/min)	BP static (mmHg)	BP IBM (mmHg)	Peak O_2_ desaturation (%)
BHD 1	95	146	241	66	51	131	162	–
BHD 2	243	156	399	63	71	89	131	55
BHD 3	150	98	248	67	58	93	114	91
BHD 4	434	103	331	70	52	113	143	48
BHD 5	131	102	233	60	65	103	118	72
BHD 6	139	179	318	54	49	111	122	80
BHD 7	248	161	409	82	53	105	102	54
BHD 8	133	57	190	41	42	130	158	91
BHD 9	159	162	321	92	85	114	141	48
BHD 10	107	112	219	55	58	110	144	87
Mean ± SD	173.6 ± 75.4	127.6 ± 38.7	301.2 ± 88.0	64.9 ± 14.5	58.3 ± 12.5	109.9 ± 13.6	133.5 ± 19.5	69.6 ± 18.5

*Note*: Oximetry data were not available for the first participant.

Abbreviations: BHD, breath‐hold diver; BP, mean blood pressure measured from the calibrated Finometer signal, calculated over the static phase of the apnoea or during the period of IBMs; HR, heart rate, calculated over the static phase of the apnoea or during the period of IBMs; IBM, inspiratory breathing movement.

**FIGURE 8:**
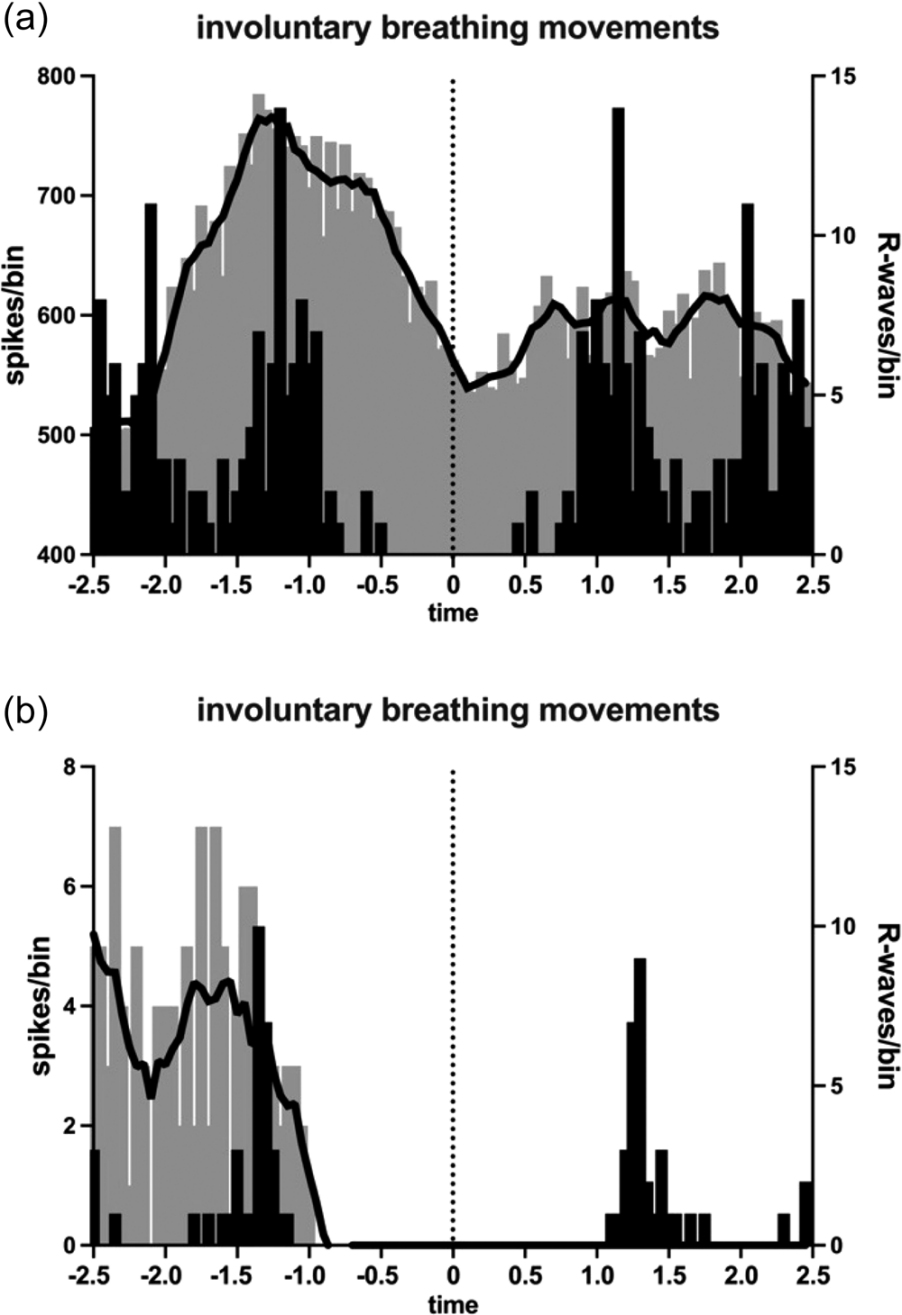
Cross‐correlation histograms (grey bars) between two intrafascicular sites of the right vagus nerve and the ECG, together with autocorrelation histograms of the ECG (black bars), recorded from a 39‐year‐old (BHD 7) (a) and a 36‐year‐old (BHD 8) (b) breath‐hold diver during the final stages of a maximal apnoea in which involuntary breathing movements occurred. Note that the primary modulation of vagal activity in both examples occurred prior to the R‐wave. Bin width = 50 ms for both sets of histograms. Time 0 corresponds to the triggering R‐wave of the ECG. The continuous black lines are the smoothed polynomials fitted to the cross‐correlation histograms to illustrate better the cardiac rhythmicity of the vagal activity.

Although there was a clear respiratory component to the modulation of vagal activity during the IBMs, it was also apparent that there were cardiac changes during these inspiratory movements. As shown in Figure 8 for two BHDs, most of the vagal neural activity occurred before the R‐wave, suggesting that it was related to inspiratory‐mediated changes in atrial stretch associated with the inspiratory efforts; because these efforts were made against a closed glottis, there was no net airflow, hence no net change in intrathoracic volume.

When cardiac modulation indices were compared during the static phase of the maximal apnoea and during the later stage when IBMs were present, there was no difference in the magnitude of modulation: 22.5% ± 19.7% versus 26.4% ± 29.0% (*p* = 0.9929; Mann–Whitney *U*‐test). Moreover, despite the very high chemical drive during a maximal apnoea, especially during the later stages when the participants were trying to suppress their urge to breathe (as evidenced by the IBMs), the magnitude of the cardiac modulation was no higher than at baseline (20.6% ± 18.3%), either during the static phase (*p* = 0.9170, Mann–Whitney *U*‐test) or during the IBMs (*p* = 0.6607, Mann–Whitney *U*‐test).

## 4 DISCUSSION

This study extends our previous work on microelectrode recordings from intrafascicular sites within the human cervical vagus nerve in healthy participants. We specifically targeted sites displaying cardiac rhythmicity to investigate potential differences between healthy control subjects and elite BHDs. Researchers in our laboratory have undertaken extensive work on the latter, trying to understand the physiological adaptations that allow BHDs to hold their breath for such a long time. As outlined above, there are no obvious differences in baseline physiological parameters of divers that set them apart, at least in terms of resting heart rate, blood pressure or vascular resistance (Heusser et al., 2009), nor differences in structure or function of the heart at rest (Kelly et al., 2022). Moreover, there are no differences in sympathetic outflow to the muscle vascular bed (Dujic et al., 2008), but until now there has been no direct measure of parasympathetic activity to and from the heart. Nonetheless, given the above, it is perhaps not surprising that the primary metric we assessed (the magnitude of cardiac modulation of multi‐unit activity) was not significantly different between the divers and control subjects.

We assessed cardiac and respiratory modulation of vagal activity during tidal breathing and slow‐deep breathing. Given that one cannot assess respiratory modulation during an apnoea, only cardiac modulation was measured during a 40 s inspiratory‐capacity apnoea, a 40 s end‐expiratory apnoea and, for the BHDs, during a maximal apnoea performed at inspiratory capacity. Holding one's breath at maximal lung inflation causes an increase in intrathoracic pressure by the elastic recoil of the lungs and chest wall against the closed glottis. Unlike a Valsalva manoeuvre, there is no active expiratory muscle activity and no ongoing activity in the inspiratory pump muscles; only the laryngeal adductors are active to keep the glottis closed. The increase in intrathoracic pressure, which has been measured as 44 ± 4 cmH_2_O in young healthy participants (Colebatch et al., 1979), increases the transmural pressure across the heart and unloads the low‐pressure baroreceptors, especially those in the compliant right atrium, leading to a sustained increase in muscle sympathetic nerve activity (Macefield & Wallin, 1995b; Macefield et al., 2006); it is the high intrathoracic pressure rather than the high lung volume that is responsible for the sympathoexcitation (Macefield, 1998a). Conversely, an end‐expiratory apnoea causes an increase in MSNA only once an adequate level of chemoreceptor drive is achieved, with progressively larger bursts of MSNA occurring as the asphyxic break‐point is approached (Halliwell & Minson, 2002; Halliwell et al., 2003; Marshall, 1994; Saito et al., 1988; Somers et al., 1989a, 1989b).

Although we have previously quantified the cardiac and respiratory modulation of multi‐unit activity within the human vagus nerve, we do know that there is a very wide variation in the magnitudes of modulation across sites within the same fascicle, within the same nerve (left or right vagus) and within the same participant (Patros et al., 2022). Modulation indices for the present cohort of control subjects (Table 2) at rest were comparable to those we had reported previously in a different cohort of healthy participants at rest, 23.4% and 44.1% for cardiac and respiratory modulation, respectively (Patros et al., 2022). And although there was no difference in the magnitude of cardiac modulation between the BHDs and control subjects at rest (tidal breathing), during slow‐deep breathing or an inspiratory‐capacity or end‐expiratory apnoea, we would have expected modulation to have changed during the maximal apnoeas performed by the BHDs, particularly during the phase when chemical drive to breathe is so elevated that involuntary breathing movements are occurring. These inspiratory efforts against a closed glottis would have caused large changes in intrathoracic pressure without any net change in thoracic volume. One would expect that the phasic reductions in intrathoracic pressure would lead to changes in transmural pressure across the heart, particularly across the more compliant atria and pulmonary veins, and affect the firing of cardiac mechanoreceptors, especially of the low‐pressure baroreceptors that are sensitive to volume changes. Using cardiac MRI, we have previously shown that during maximal inspiratory apnoea, with inspired total lung capacity even higher than total lung capacity with ‘lung packing’, the more compressible right heart is predominantly affected, with reduced right heart volumes (Batinic et al., 2011). The occurrence of IBMs is followed by augmentation of stroke volume and renormalization of the cardiac output via increased inferior vena caval flow (Palada et al., 2008). Furthermore, we have found that an increase in the IBMs transiently increases the cerebral blood volume (Dujic et al., 2009). The cardiac‐locked bursts during the involuntary breathing movements preceded the R‐wave, hinting at their likely identity as reflecting the activity of atrial receptors, as will be discussed below.

It needs to be emphasized that our multi‐unit recordings of neural activity from the right vagus nerve contain discharges from both sensory and motor parasympathetic axons, but it is not unreasonable to conclude that those sites expressing cardiac modulation do supply the heart and its associated nearby vessels. As we had recently described, it is only by extracting action potentials from individual axons within the vagus nerve that one can attempt to classify them as sensory or motor (Farmer et al., 2025). At the very least, single‐unit recordings allow one to identify the axon as myelinated or unmyelinated; extracellular potentials recorded from a myelinated axon with a metal microelectrode have a dominant positive‐going spike, whereas unmyelinated axons generate negative‐going spikes (Inglis et al., 1996; Macefield, 1998b; Macefield et al., 1994; Vallbo, 1976, 2018). In vivo and in vitro studies have demonstrated that positive‐going spikes are generated when a microelectrode impales the myelin sheath; these increase in amplitude as the axonal membrane is approached and become negative‐going when the microelectrode is withdrawn from the myelin (David et al., 1992, 1993). We could not measure conduction velocities, because this would require inserting a second (stimulating) microelectrode some distance from the recording microelectrode, but we are currently measuring conduction velocities of vagal axons in patients with implanted vagus nerve stimulators (Patros et al., 2024).

It is also important to acknowledge that the identity of an axon as afferent or efferent, in the absence of data that would require invasive studies of the heart, can be inferred only on the basis of behavioural criteria. In other words, does this axon behave like an atrial receptor or like a cardioinhibitory motor axon, for example? We know that most of the axons within the cervical vagus nerve are sensory (Ottaviani & Macefield, 2022), and we had argued previously that this would mean that most of the recordings we obtain are from afferents (Farmer et al., 2025). Our single‐unit analysis found very few myelinated axons that behaved like cardioinhibitory (motor) axons (Farmer et al., 2025), hence it is most likely that the myelinated axons recorded from the right vagus nerve that exhibit cardiac rhythmicity are mechanoreceptors located within the atria and pulmonary veins. These endings fire with a clear cardiac rhythmicity (Brown, 1965; Coleridge et al., 1964; Paintal, 1953a, 1953b; Thorén et al., 1977; Whitteridge, 1948), with many of these also being modulated by respiration (Coleridge et al., 1964; Paintal, 1953a, 1973).

Although early work supported the idea that there were three types of atrial receptor, type A, type B and intermediate, that fired early, late or across the cardiac cycle, respectively, it is more likely that there is only one type of atrial mechanoreceptor and that its firing pattern depends on its location and orientation within the heart (Arndt et al., 1974; Kappagoda et al., 1976, 1977). Of course, confirmation of the identity of an atrial receptor would have to rely on invasive studies of the heart or at least discrete mechanical stimulation of the receptive field with a balloon catheter inserted into the atrium or pulmonary veins (Farmer et al., 2025). Moreover, myelinated ventricular receptors fire during systole, that is, at the same time at which ‘type B’ atrial receptors also fire, hence differentiating the two remains problematic, not the least being that mechanoreceptors in the coronary vessels can discharge in a similar fashion (al‐Timman et al., 1993; Brown, 1965; Drinkhill et al., 1993; Moore et al., 2022). Nevertheless, both ventricular and coronary mechanoreceptors are rare (Chapman & Pearce, 1959; Coleridge et al., 1964; Drinkhill et al., 1993; Zucker & Gilmore, 1977), hence the most parsimonious interpretation is that the multi‐unit recordings we have obtained, dominated by the cardiac‐locked discharges of myelinated axons, primarily reflect the activity of atrial baroreceptors. Our cross‐correlation analyses showed that the peak modulation of firing preceded, straddled or followed the R‐wave, no doubt reflecting the types of sensory axons making up each recording site.

Studies in experimental animals have also shown that unmyelinated endings can be found throughout the heart (in the atria, ventricles, coronary arteries and great vessels); they are not spontaneously active at rest but are sensitive to the chemical environment of the tissues and more extreme pressures (Coleridge et al., 1973, 1964; Drinkhill et al., 1993; Zucker, 1986). In this regard, the recording illustrated in Figure 1, dominated by cardiac‐locked phasic bursts of negative‐going spikes, is interesting. This multi‐unit recording was obtained in a young BHD who had earlier experienced a suspected psychogenic vasovagal event, characterized by several asystolic pauses and marked hypotension (this case was described briefly by Patros et al., 2025). The phasic discharges were modulated by respiration, increasing during inspiration, and this was augmented during slow‐deep breathing; volume changes within the atria would be increased with the exaggerated inspiratory reductions in intrathoracic pressure, leading to an increase in venous return, hence an increase in volume of the atria (and of blood vessels associated with the lungs). But the fact that the site was dominated by unmyelinated axons (i.e., negative‐going spikes) is of itself unusual, given what we have written above about the relative scarcity of unmyelinated cardiac receptors exhibiting spontaneous activity. Indeed, perhaps this discharge was somewhat pathophysiological, given that the blood pressure of the participant was still low when this recording was obtained (although this participant, BHD 2, did have a normally low systolic pressure at rest; Table 2).

The double bursts illustrated in Figure 3, recorded during the same session in the same diver, are also interesting. This multi‐unit site, dominated by myelinated axons, was unusual in that two phasic bursts were evoked during each cardiac cycle; aligning the continuous non‐invasive blood pressure recording to the neural recording demonstrated that the first burst was associated with systole and the second with the dichrotic notch. The latter corresponds to closure of the aortic valve following ejection of blood from the left ventricle (Abushouk et al., 2023). As such, the first burst might reflect mechanoreceptor activity associated with ventricular contraction against the closed valve and the second with its opening. Could this recording be dominated by stretch receptor activity in the papillary muscles that hold the valve closed? Again, in the absence of direct evidence of the receptive fields of these endings, any discussion is pure speculation, but given that neither atrial nor ventricular mechanoreceptors fire with two bursts in the cardiac cycle, our suggestion is not unreasonable. However, we are not aware of any studies to have characterized the firing properties of mechanoreceptors associated with the papillary muscles or chordae tendinae, or even whether such endings exist.

### 4.1 Limitations

This was an ambitious study to use tungsten microelectrodes to record multi‐unit activity from the right vagus nerve during maximal apnoea in elite BHDs. We used ultrasound to guide the microelectrode into the nerve and confirmed that the tip was located within a fascicle of the nerve. Remarkably, we managed to obtain stable recordings despite the BHDs performing several practice trials while the microelectrode remained in situ. We always used ultrasound to check that the microelectrode tip was still in the nerve following an attempt, repositioning the tip if necessary, and we are convinced that the recordings reported herein were obtained from fascicles of the vagus nerve. As noted above, we obtained fewer recording sites within the divers than within the control participants; for the latter, we had the luxury of being able to spend time exploring the nerve to find sites exhibiting cardiac modulation, whereas for the divers we were keen to proceed with the experiment as soon as we found a suitable site. Moreover, the BHDs wanted to practice their apnoeas, and they were aware that they must do this without causing large neck movements that could dislodge the microelectrode, hence the requirement not to undertake ‘lung‐packing’. We applied a gel ice‐pack to their faces immediately prior to the maximal apnoea to increase trigeminal inputs and thereby evoke the diving reflex, a feature of which is vagally‐mediated bradycardia (James & de Burgh Daly, 1972; Schagatay & Holm, 1996).

Although we obtained data from only 10 BHDs and believe these recordings are representative of a larger sample, there is no doubt that having more data might well increase our statistical power to reveal potential differences in cardiac‐related neural signalling between the divers and control participants. What leads us away from this is that, as noted in the Introduction, all non‐invasive and invasive physiological studies conducted on these elite divers by researchers in our laboratory over the years have failed to find marked differences in baseline physiology, aside from a larger spleen and total lung capacity in the BHDs, that can explain their remarkable capacity to hold their breath for so long (for review, see Bain et al., 2018b). Although the ages of the participants were not matched (the controls were younger) and most were male, they were all healthy. Clearly, training to suppress the urge to breathe during extreme hypoxaemia is key; it is this top‐down control of respiration that allows the body to do what it is good at, regulating diverse physiological systems to allow it to survive in the face of extreme stress.

Finally, a very important caveat is that we do not know whether the multi‐unit sites in which we detected cardiac‐modulated activity are composed of afferent or efferent axons or both. For obvious reasons, we are limited in what we can do in awake humans, but using the principle of parsimony, it is more likely that the activity we have recorded is dominated by afferent axons (most of the vagus nerve is composed of sensory fibres; Ottaviani & Macefield, 2022). As noted above, a single‐unit analysis would provide more security in defining an axon as afferent or efferent, but again, this is limited to a behavioural interpretation and comparison with what is known about the firing properties of single vagal axons studies in anaesthetized and surgically reduced experimental animals.

## 5 CONCLUSION

We have characterized the cardiac and respiratory rhythmicity of multi‐unit recordings obtained from the right cervical vagus nerve in elite BHDs for the first time. Surprisingly, there were no differences in the magnitude of cardiac modulation between the divers at rest (tidal breathing), during slow‐deep breathing, during an inspiratory‐capacity apnoea or during an end‐expiratory apnoea. There were also no differences in the magnitude of respiratory modulation between the two groups at rest or during slow‐deep breathing. Moreover, the magnitude of cardiac modulation during the static phase of a maximal apnoea in the BHDs was not augmented during the static phase of the apnoea, when there is no phasic respiratory input, or during the later stages of the apnoea, when involuntary breathing movements occur prior to reaching the asphyxic break‐point. Given that one would expect any cardioinhibitory drive to be reduced during the early stages of an apnoea, in which heart rate and blood pressure are increasing (the latter owing to a sympathetically mediated increase in vasoconstriction), an increase in cardioinhibitory drive might be expected to occur prior to the asphyxic break‐point, when periods of bradycardia may or may not be present.

## AUTHOR CONTRIBUTIONS

Vaughan G. Macefield and Zeljko Dujic helped with the conception or design of the work. Vaughan G. Macefield helped with the acquisition, analysis or interpretation of data for the work; drafted the work and revised it critically for important intellectual content. Anthony R. Bain, Matthew I. Badour, Marko Kumric, Ivan Drvis, Otto F. Barak, Josko Bozic, Zeljko Dujic helped with the acquisition, analysis or interpretation of data for the work; drafting the work and revising it critically for important intellectual content. All authors approved the final version of the manuscript and agree to be accountable for all aspects of the work in ensuring that questions related to the accuracy or integrity of any part of the work are appropriately investigated and resolved. All persons designated as authors qualify for authorship, and all those who qualify for authorship are listed.

## CONFLICT OF INTEREST

None declared.

## Data Availability

Curated data are available on reasonable request.

## References

[eph13910-bib-0001] Abushouk, A. , Kansara, T. , Abdelfattah, O. , Badwan, O. , Hariri, E. , Chaudhury, P. , & Kapadia, S. R. (2023). The dicrotic notch: Mechanisms, characteristics, and clinical correlations. Current Cardiology Reports, 25(8), 807–816. 37493873 10.1007/s11886-023-01901-x

[eph13910-bib-0002] al‐Timman, J. K. , Drinkhill, M. J. , & Hainsworth, R. (1993). Reflex responses to stimulation of mechanoreceptors in the left ventricle and coronary arteries in anaesthetized dogs. The Journal of Physiology, 472(1), 769–783. 8145171 10.1113/jphysiol.1993.sp019972 PMC1160512

[eph13910-bib-0003] Arndt, J. O. , Brambring, P. , Hindorf, K. , & Röhnelt, M. (1974). The afferent discharge pattern of atrial mechanoreceptors in the cat during sinusoidal stretch of atrial strips in situ. The Journal of Physiology, 240(1), 33–52. 4854642 10.1113/jphysiol.1974.sp010597 PMC1330979

[eph13910-bib-0004] Bain, A. R. , Ainslie, P. N. , Hoiland, R. L. , Barak, O. F. , Drvis, I. , Stembridge, M. , MacLeod, D. M. , McEneny, J. , Stacey, B. S. , Tuaillon, E. , Marchi, N. , Fayd'Herbe De Maudave, A. , Dujic, Z. , MacLeod, D. B. , & Bailey, D. M. (2018). Competitive apnea and its effect on the human brain: Focus on the redox regulation of blood‐brain barrier permeability and neuronal‐parenchymal integrity. Federation of American Societies for Experimental Biology Journal, 32(4), 2305–2314. 29191963 10.1096/fj.201701031R

[eph13910-bib-0005] Bain, A. R. , Ainslie, P. N. , Hoiland, R. L. , Willie, C. K. , MacLeod, D. B. , Madden, D. , Maslov, P. Z. , Drviš, I. , & Dujić, Ž. (2016). Role of cerebral blood flow in extreme breath holding. Translational Neuroscience, 7(1), 12–16. 28123816 10.1515/tnsci-2016-0003 PMC5017590

[eph13910-bib-0006] Bain, A. R. , Drvis, I. , Dujic, Z. , MacLeod, D. B. , & Ainslie, P. N. (2018). Physiology of static breath holding in elite apneists. Experimental Physiology, 103(5), 635–651. 29512224 10.1113/EP086269

[eph13910-bib-0007] Bakovic, D. , Valic, Z. , Eterovic, D. , Vukovic, I. , Obad, A. , Marinovic‐Terzic, I. , & Dujic, Z. (2003). Spleen volume and blood flow response to repeated breath‐hold apneas. Journal of Applied Physiology, 95(4), 1460–1466. 12819225 10.1152/japplphysiol.00221.2003

[eph13910-bib-0008] Batinic, T. , Utz, W. , Breskovic, T. , Jordan, J. , Schulz‐Menger, J. , Jankovic, S. , Dujic, Z. , & Tank, J. (2011). Cardiac magnetic resonance imaging during pulmonary hyperinflation in apnea divers. Medicine and Science in Sports and Exercise, 43(11), 2095–2101. 21552160 10.1249/MSS.0b013e31821ff294

[eph13910-bib-0009] Berenbrink, M. (2021). The role of myoglobin in the evolution of mammalian diving capacity—The August Krogh principle applied in molecular and evolutionary physiology. Comparative Biochemistry and Physiology‐ Part A, Molecular & Integrative Physiology, 252, 110843. 10.1016/j.cbpa.2020.110843 33181325

[eph13910-bib-0010] Breskovic, T. , Ivancev, V. , Banic, I. , Jordan, J. , & Dujic, Z. (2010). Peripheral chemoreflex sensitivity and sympathetic nerve activity are normal in apnea divers during training season. Autonomic Neuroscience, 154(1‐2), 42–47. 19926535 10.1016/j.autneu.2009.11.001

[eph13910-bib-0011] Breskovic, T. , Valic, Z. , Lipp, A. , Heusser, K. , Ivancev, V. , Tank, J. , Dzamonja, G. , Jordan, J. , Shoemaker, J. K. , Eterovic, D. , & Dujic, Z. (2010). Peripheral chemoreflex regulation of sympathetic vasomotor tone in apnea divers. Clinical Autonomic Research, 20(2), 57–63. 19820987 10.1007/s10286-009-0034-1

[eph13910-bib-0012] Brown, A. M. (1965). Mechanoreceptors in or near the coronary arteries. The Journal of Physiology, 177(2), 203–214. 14301021 10.1113/jphysiol.1965.sp007586 PMC1357239

[eph13910-bib-0013] Chapman, K. M. , & Pearce, J. W. (1959). Vagal afferents in the monkey. Nature, 184(4694), 1237–1238. 10.1038/1841237a0 13809236

[eph13910-bib-0014] Colebatch, H. J. H. , Greaves, I. A , & Ng, C. K. Y. (1979). Exponential analysis of elastic recoil and aging in healthy males and females. Journal of Applied Physiology, 47(4), 683–691. 511674 10.1152/jappl.1979.47.4.683

[eph13910-bib-0015] Coleridge, H. M. , Coleridge, J. C. , Dangel, A. , Kidd, C. , Luck, J. C. , & Sleight, P. (1973). Impulses in slowly conducting vagal fibers from afferent endings in the veins, atria, and arteries of dogs and cats. Circulation Research, 33(1), 87–97. 4587825 10.1161/01.res.33.1.87

[eph13910-bib-0016] Coleridge, H. M. , Coleridge, J. C. , & Kidd, C. (1964). Cardiac receptors in the dog, with particular reference to two types of afferent ending in the ventricular wall. The Journal of Physiology, 174(3), 323–339. 14232396 10.1113/jphysiol.1964.sp007490 PMC1368933

[eph13910-bib-0017] David, G. , Barrett, J. H. , & Barrett, E. F. (1992). Evidence that action potentials activate an internodal potassium conductance in lizard myelinated axons. The Journal of Physiology, 445(1), 277–301. 1501136 10.1113/jphysiol.1992.sp018924 PMC1179982

[eph13910-bib-0018] David, G. , Barrett, J. H. , & Barrett, E. F. (1993). Activation of internodal potassium conductance in rat myelinated axons. The Journal of Physiology, 472(1), 177–202. 8145140 10.1113/jphysiol.1993.sp019942 PMC1160482

[eph13910-bib-0019] Dempsey, J. A. , Sheel, A. W. , St. Croix, C. M. , & Morgan, B. (2002). Respiratory influences on sympathetic vasomotor outflow in humans. Respiratory Physiology & Neurobiology, 130(1), 3–20. 12380012 10.1016/s0034-5687(01)00327-9

[eph13910-bib-0020] Drinkhill, M. J. , Moore, J. , & Hainsworth, R. (1993). Afferent discharges from coronary arterial and ventricular receptors in anaesthetized dogs. The Journal of Physiology, 472(1), 785–799. 8145172 10.1113/jphysiol.1993.sp019973 PMC1160513

[eph13910-bib-0021] Dujic, Z. , Ivancev, V. , Heusser, K. , Dzamonja, G. , Palada, I. , Valic, Z. , Tank, J. , Obad, A. , Bakovic, D. , Diedrich, A. , Joyner, M. J. , & Jordan, J. (2008). Central chemoreflex sensitivity and sympathetic neural outflow in elite breath‐hold divers. Journal of Applied Physiology, 104(1), 205–211. 17991789 10.1152/japplphysiol.00844.2007

[eph13910-bib-0022] Dujic, Z. , Uglesic, L. , Breskovic, T. , Valic, Z. , Heusser, K. , Marinovic, J. , Ljubkovic, M. , & Palada, I. (2009). Involuntary breathing movements improve cerebral oxygenation during apnea struggle phase in elite divers. Journal of Applied Physiology, 107(6), 1840–1846. 19850736 10.1152/japplphysiol.00334.2009

[eph13910-bib-0023] Dzamonja, G. , Tank, J. , Heusser, K. , Palada, I. , Valic, Z. , Bakovic, D. , Obad, A. , Ivancev, V. , Breskovic, T. , Diedrich, A. , Luft, F. C. , Dujic, Z. , & Jordan, J. (2010). Glossopharyngeal insufflation induces cardioinhibitory syncope in apnea divers. Clinical Autonomic Research, 20(6), 381–384. 20623312 10.1007/s10286-010-0075-5

[eph13910-bib-0024] Eckberg, D. L. , Nerhed, C. , & Wallin, B. G. (1985). Respiratory modulation of muscle sympathetic and vagal cardiac outflow in man. The Journal of Physiology, 365(1), 181–196. 4032310 10.1113/jphysiol.1985.sp015766 PMC1192996

[eph13910-bib-0025] Farmer, D. G. S. , Patros, M. , Ottaviani, M. M. , Dawood, T. , Kumric, M. , Bozic, J. , Badour, M. I. , Bain, A. R. , Barak, O. F. , Dujic, Z. , & Macefield, V. G. (2025). Firing properties of single axons with cardiac rhythmicity in the human cervical vagus nerve. The Journal of Physiology, 603, 1941–1958. 39320231 10.1113/JP286423 PMC11955867

[eph13910-bib-0026] Godfrey, S. , & Campbell, E. J. (1969). Mechanical and chemical control of breath holding. Quarterly Journal of Experimental Physiology and Cognate Medical Sciences, 54, 117–128. 5193728 10.1113/expphysiol.1969.sp002011

[eph13910-bib-0027] Godfrey, S. , Edwards, R. H. , & Warrell, D. A. (1969). The influence of lung shrinkage on breath holding time. Quarterly Journal of Experimental Physiology and Cognate Medical Sciences, 54, 129–140. 5193890 10.1113/expphysiol.1969.sp002012

[eph13910-bib-0028] Halliwell, J. R , & Minson, C. T. (2002). Effect of hypoxia on arterial baroreflex control of heart rate and muscle sympathetic nerve activity in humans. Journal of Applied Physiology, 93(3), 857–864. 12183478 10.1152/japplphysiol.01103.2001

[eph13910-bib-0029] Halliwell, J. R. , Morgan, B. , & Charkoudian, N. (2003). Peripheral chemoreflex and baroreflex interaction in cardiovascular regulation in humans. The Journal of Physiology, 552(1), 295–302. 12897165 10.1113/jphysiol.2003.050708 PMC2343329

[eph13910-bib-0030] Hardy, J. C. , Gray, K. , Whisler, S. , & Leuenberger, U. (1994). Sympathetic and blood pressure responses to voluntary apnea are augmented by hypoxemia. Journal of Applied Physiology, 77(5), 2360–2365. 7868456 10.1152/jappl.1994.77.5.2360

[eph13910-bib-0031] Heusser, K. , Dzamonja, G. , Tank, J. , Palada, I. , Valic, Z. , Bakovic, D. , Obad, A. , Ivancev, V. , Breskovic, T. , Diedrich, A. , Joyner, M. J. , Luft, F. C. , Jordan, J. , & Dujic, Z. (2009). Cardiovascular regulation during apnea in elite divers. Hypertension, 53(4), 719–724. 19255361 10.1161/HYPERTENSIONAHA.108.127530

[eph13910-bib-0032] Inglis, J. T. , Leeper, J. B. , Burke, D. , & Gandevia, S. C. (1996). Morphology of action potentials recorded from human nerves using microneurography. Experimental Brain Research, 110(2), 308–314. 8836694 10.1007/BF00228561

[eph13910-bib-0033] Ivancev, V. , Palada, I. , Valic, Z. , Obad, A. , Bakovic, D. , Dietz, N. M. , Joyner, M. J. , & Dujic, Z. (2007). Cerebrovascular reactivity to hypercapnia is unimpaired in breath‐hold divers. The Journal of Physiology, 582(2), 723–730. 17412771 10.1113/jphysiol.2007.128991 PMC2075341

[eph13910-bib-0034] James, J. E. , & de Burgh Daly, M. (1972). Some mechanisms involved in the cardiovascular adaptations to diving. Symposia of the Society for Experimental Biology, 216, 313–341. 4581876

[eph13910-bib-0035] Kappagoda, C. T. , Linden, R. J. , & Mary, D. A. (1976). Atrial receptors in the cat. The Journal of Physiology, 262(2), 431–446. 994043 10.1113/jphysiol.1976.sp011603 PMC1307651

[eph13910-bib-0036] Kappagoda, C. T. , Linden, R. J. , & Mary, D. A. (1977). Atrial receptors in the dog and rabbit. The Journal of Physiology, 272(3), 799–815. 592216 10.1113/jphysiol.1977.sp012074 PMC1353656

[eph13910-bib-0037] Kelly, T. , Brown, C. , Bryant‐Ekstrand, M. , Lord, R. , Dawkins, T. , Drane, A. , Futral, J. E. , Barak, O. , Dragun, T. , Stembridge, M. , Spajić, B. , Drviš, I. , Duke, J. W. , Ainslie, P. N. , Foster, G. E. , Dujic, Z. , & Lovering, A. T. (2022). Blunted hypoxic pulmonary vasoconstriction in apnoea divers. Experimental Physiology, 107(11), 1225–1240. 35993480 10.1113/EP090326

[eph13910-bib-0038] Kelman, G. R. , & Wann, K. T. (1971). Mechanical and chemical control of breath holding. Quarterly Journal of Experimental Physiology and Cognate Medical Sciences, 56, 92–100. 5206674 10.1113/expphysiol.1971.sp002111

[eph13910-bib-0039] Kiviniemi, A. M. , Breskovic, T. , Uglesic, L. , Kuch, B. , Maslov, P. Z. , Sieber, A. , Seppänen, T. , Tulppo, M. P. , & Dujic, Z. (2012). Heart rate variability during static and dynamic breath‐hold dives in elite divers. Autonomic Neuroscience, 169(2), 95–101. 22682754 10.1016/j.autneu.2012.05.004

[eph13910-bib-0040] Lemaître, F. , Buchheit, M. , Joulia, F. , Fontanari, P. , & Tourny‐Chollet, C. (2008). Static apnea effect on heart rate and its variability in elite breath‐hold divers. Aviation Space and Environmental Medicine, 79(2), 99–104. 18309906 10.3357/asem.2142.2008

[eph13910-bib-0041] Leuenberger, U. , Jacob, E. , Sweer, L. , Waravdekar, N. , Zwillich, C. , & Sinoway, L. (1995). Surges of muscle sympathetic activity during obstructive apnea are linked to hypoxemia. Journal of Applied Physiology, 79(2), 581–588. 7592221 10.1152/jappl.1995.79.2.581

[eph13910-bib-0042] Macefield, V. G. (1998a). Sustained activation of muscle sympathetic outflow during lung inflation depends on a high intrathoracic pressure. Journal of the Autonomic Nervous System, 68(3), 135–139. 9626939 10.1016/s0165-1838(97)00129-x

[eph13910-bib-0043] Macefield, V. G. (1998b). Spontaneous and evoked ectopic discharges recorded from single human axons. Muscle & Nerve, 21(4), 461–468. 9533780 10.1002/(sici)1097-4598(199804)21:4<461::aid-mus4>3.0.co;2-7

[eph13910-bib-0044] Macefield, V. G , & Wallin, B. G. (1995a). Modulation of muscle sympathetic activity during spontaneous and artificial ventilation and apnoea in humans. Journal of the Autonomic Nervous System, 53(2‐3), 137–147. 7560750 10.1016/0165-1838(94)00173-h

[eph13910-bib-0045] Macefield, V. G. , Gandevia, S. C. , & Henderson, L. A. (2006). Neural sites involved in the sustained increase in muscle sympathetic nerve activity induced by inspiratory‐capacity apnea—A fMRI study. Journal of Applied Physiology, 100(1), 266–273. 16123207 10.1152/japplphysiol.00588.2005

[eph13910-bib-0046] Macefield, V. G. , & Wallin, B. G. (1995b). Effects of static lung inflation on sympathetic activity in human muscle nerves at rest and during asphyxia. Journal of the Autonomic Nervous System, 53(2‐3), 148–156. 7560751 10.1016/0165-1838(94)00174-i

[eph13910-bib-0047] Macefield, V. G. , Wallin, B. G. , & Vallbo, A. B. (1994). The discharge behaviour of single vasoconstrictor motoneurones in human muscle nerves. The Journal of Physiology, 481(3), 799–809. 7707244 10.1113/jphysiol.1994.sp020482 PMC1155919

[eph13910-bib-0048] Marshall, J. M. (1994). Peripheral chemoreceptors and cardiovascular regulation. Physiological Reviews, 74(3), 543–594. 8036247 10.1152/physrev.1994.74.3.543

[eph13910-bib-0049] Moore, J. P. , Simpson, L. L. , & Drinkhill, M. J. (2022). Differential contributions of cardiac, coronary and pulmonary artery vagal mechanoreceptors to reflex control of the circulation. The Journal of Physiology, 600(18), 4069–4087. 35903901 10.1113/JP282305 PMC9544715

[eph13910-bib-0050] Morgan, B. , Crabtree, D. C. , Palta, M. , & Skatrud, J. B. (1995). Combined hypoxia and hypercapnia evokes long‐lasting sympathetic activation in humans. Journal of Applied Physiology, 79(1), 205–213. 7559221 10.1152/jappl.1995.79.1.205

[eph13910-bib-0051] Muenter Swift, N. , Cutler, M. , Fadel, P. , Wasmund, W. , Ogoh, S. , Keller, D. , Raven, P. , & Smith, M. (2003). Carotid baroreflex function during and following voluntary apnea in humans. American Journal of Physiology‐ Heart and Circulatory Physiology, 285(6), H2411–H2419. 12893634 10.1152/ajpheart.00139.2003

[eph13910-bib-0052] Ottaviani, M. M. , & Macefield, V. G. (2022). Structure and functions of the vagus nerve in mammals. Comprehensive Physiology, 12(4), 3989–4037. 35950655 10.1002/cphy.c210042

[eph13910-bib-0053] Ottaviani, M. M. , Wright, L. , Dawood, T. , & Macefield, V. G. (2020). In‐vivo recordings from the human vagus nerve using ultrasound‐guided microneurography. Journal of Physiology, 598(17), 3569–3576. 32538473 10.1113/JP280077

[eph13910-bib-0054] Paintal, A. S. (1953a). A study of right and left atrial receptors. The Journal of Physiology, 120(4), 596–610. 13070230 10.1113/jphysiol.1953.sp004920 PMC1366006

[eph13910-bib-0055] Paintal, A. S. (1953b). The conduction velocities of respiratory and cardiovascular afferent fibres in the vagus nerve. The Journal of Physiology, 121(2), 341–359. 13085339 10.1113/jphysiol.1953.sp004950 PMC1366079

[eph13910-bib-0056] Paintal, A. S. (1963). Vagal afferent fibres. Ergebnisse Der Physiologie, Biologischen Chemie Und Experimentellen Pharmakologie, 52(1), 74–156. 14281179

[eph13910-bib-0057] Paintal, A. S. (1973). Vagal sensory receptors and their reflex effects. Physiological Reviews, 53(1), 159–227. 4568412 10.1152/physrev.1973.53.1.159

[eph13910-bib-0058] Palada, I. , Bakovic, D. , Valic, Z. , Obad, A. , Ivancev, V. , Eterovic, D. , Shoemaker, J. K. , & Dujic, Z. (2008). Restoration of hemodynamics in apnea struggle phase in association with involuntary breathing movements. Respiratory Physiology & Neurobiology, 161(2), 174–181. 18337193 10.1016/j.resp.2008.01.008

[eph13910-bib-0059] Patros, M. , Farmer, D. G. S. , Moneghetti, K. , Ottaviani, M. M. , Sivathamboo, S. , Simpson, H. D. , O'Brien, T. J. , & Macefield, V. G. (2024). First‐in‐human microelectrode recordings from the vagus nerve during clinical vagus nerve stimulation. Epilepsia Open, 9(6), 2522–2527. 39465627 10.1002/epi4.13083 PMC11633718

[eph13910-bib-0060] Patros, M. , Farmer, D. G. S. , Ottaviani, M. M. , Dawood, T. , Kumric, M. , Bozic, J. , Badour, M. I. , Bain, A. R. , Drvis, I. , Barak, O. F. , Dujic, Z. , & Macefield, V. G. (2025). Risk of bradycardia and asystole during microelectrode recordings from the human vagus nerve. Clinical Autonomic Research, 35(2), 341–345. 39673646 10.1007/s10286-024-01101-9

[eph13910-bib-0061] Patros, M. , Ottaviani, M. M. , Wright, L. , Dawood, T. , & Macefield, V. G. (2022). Quantification of cardiac and respiratory modulation of axonal activity in the human vagus nerve. The Journal of Physiology, 600(13), 3113–3126. 35524982 10.1113/JP282994

[eph13910-bib-0062] Saito, M. , Mano, T. , Iwase, S. , Koga, K. , Abe, H. , & Yamazaki, Y. (1988). Response in muscle sympathetic activity to acute hypoxia in humans. Journal of Applied Physiology, 65(4), 1548–1552. 3182518 10.1152/jappl.1988.65.4.1548

[eph13910-bib-0063] Schagatay, E. , & Holm, B. (1996). Effects of ambient water and air temperatures on human diving bradycardia. European Journal of Applied Physiology and Occupational Physiology, 73(1–2), 1–6. 8861662 10.1007/BF00262802

[eph13910-bib-0064] Seals, D. R. , Suwarno, O. N , & Dempsey, J. A. (1990). Influence of lung volume on sympathetic nerve discharge in normal humans. Circulation Research, 67(1), 130–141. 2364488 10.1161/01.res.67.1.130

[eph13910-bib-0065] Seitz, M. J. , Brown, R. , & Macefield, V. G. (2013). Inhibition of augmented muscle vasoconstrictor drive following asphyxic apnoea in awake human subjects is not affected by relief of chemical drive. Experimental Physiology, 98(2), 405–414. 22923230 10.1113/expphysiol.2012.067421

[eph13910-bib-0066] Somers, V. K. , Mark, A. L , & Abboud, F. M. (1991). Interaction of baroreceptor and chemoreceptor reflex control of sympathetic nerve activity in normal humans. Journal of Clinical Investigation, 87(6), 1953–1957. 2040688 10.1172/JCI115221 PMC296947

[eph13910-bib-0067] Somers, V. K. , Mark, A. L. , Zavala, D. C , & Abboud, F. M. (1989a). Contrasting effects of hypoxia and hypercapnia on ventilation and sympathetic activity in humans. Journal of Applied Physiology, 67(5), 2101–2106. 2513316 10.1152/jappl.1989.67.5.2101

[eph13910-bib-0068] Somers, V. K. , Mark, A. L. , Zavala, D. C , & Abboud, F. M. (1989b). Influence of ventilation and hypocapnia on sympathetic nerve responses to hypoxia in normal humans. Journal of Applied Physiology, 67(5), 2095–2100. 2513315 10.1152/jappl.1989.67.5.2095

[eph13910-bib-0069] St Croix, C. M. , Satoh, M. , Morgan, B. , Skatrud, J. B , & Dempsey, J. A. (1999). Role of respiratory motor output in within‐breath modulation of muscle sympathetic nerve activity in humans. Circulation Research, 85(5), 457–469. 10473675 10.1161/01.res.85.5.457

[eph13910-bib-0070] Steinback, C. D. , Breskovic, T. , Dujic, Z. , & Shoemaker, J. K. (2010a). Ventilatory restraint of sympathetic activity during chemoreflex stress. American Journal of Physiology‐Regulatory, Integrative and Comparative Physiology, 299(5), R1407–R1414. 20826706 10.1152/ajpregu.00432.2010

[eph13910-bib-0071] Steinback, C. D. , Salmanpour, A. , Breskovic, T. , Dujic, Z. , & Shoemaker, J. K. (2010b). Sympathetic neural activation: An ordered affair. The Journal of Physiology, 588(23), 4825–4836. 20937711 10.1113/jphysiol.2010.195941 PMC3010149

[eph13910-bib-0072] Thoren, P. , & Jones, J. V. (1977). Characteristics of aortic baroreceptor C‐fibres in the rabbit. Acta Physiologica Scandinavica, 99(4), 448–456. 855672 10.1111/j.1748-1716.1977.tb10397.x

[eph13910-bib-0073] Thoren, P. , Noresson, E. , & Ricksten, S. E. (1979). Cardiac reflexes in normotensive and spontaneously hypertensive rats. American Journal of Cardiology, 44(5), 884–888. 386769 10.1016/0002-9149(79)90218-2

[eph13910-bib-0074] Thorén, P. , Saum, W. R. , & Brown, A. M. (1977). Characteristics of rat aortic baroreceptors with nonmedullated afferent nerve fibers. Circulation Research, 40(3), 231–237. 837469 10.1161/01.res.40.3.231

[eph13910-bib-0075] Vallbo, Å. B. (1976). Prediction of propagation block on the basis of impulse shape in single unit recordings from human nerves. Acta Physiologica Scandinavica, 97(1), 66–74. 1274638 10.1111/j.1748-1716.1976.tb10236.x

[eph13910-bib-0076] Vallbo, Å. B. (2018). Microneurography: How it started and how it works. Journal of Neurophysiology, 120(3), 1415–1427. 29924706 10.1152/jn.00933.2017

[eph13910-bib-0077] Van Den Borne, P. , Montano, N. , Narkiewitz, K. , Degaute, J. P. , Malliani, A. , Pagani, M. , & Somers, V. K. (2001). Importance of ventilation in modulating interaction between sympathetic drive and cardiovascular variability. American Journal of Physiology‐Heart and Circulatory Physiology, 280(2), H722–H729. 11158971 10.1152/ajpheart.2001.280.2.H722

[eph13910-bib-0078] Watenpaugh, D. , Muenter, N. , Wasmund, W. , Wasmund, S. , & Smith, M. (1999). Post‐apneic inhalation reverses apnea‐induced sympathoexcitation before restoration of blood oxygen levels. Sleep, 22, 435–440. 10389219

[eph13910-bib-0079] Whitteridge, D. (1948). Afferent nerve fibres from the heart and lungs in the cervical vagus. The Journal of Physiology, 107(4), 496–512. 16991830 10.1113/jphysiol.1948.sp004294 PMC1392400

[eph13910-bib-0080] Willie, C. K. , Ainslie, P. N. , Drvis, I. , MacLeod, D. B. , Bain, A. R. , Madden, D. , Maslov, P. Z. , & Dujic, Z. (2015). Regulation of brain blood flow and oxygen delivery in elite breath‐hold divers. Journal of Cerebral Blood Flow and Metabolism, 35(1), 66–73. 25370857 10.1038/jcbfm.2014.170 PMC4294396

[eph13910-bib-0081] Yao, T. , & Thoren, P. (1983). Characteristics of brachiocephalic and carotid sinus baroreceptors with non‐medullated afferents in rabbit. Acta Physiologica Scandinavica, 117(1), 1–8. 6344556 10.1111/j.1748-1716.1983.tb07172.x

[eph13910-bib-0082] Zucker, I. H. (1986). Left ventricular receptors: Physiological controllers or pathological curiosities?. Basic Research in Cardiology, 81(6), 539–557. 3545177 10.1007/BF02005179

[eph13910-bib-0083] Zucker, I. H. , & Gilmore, J. P. (1977). Cardiopulmonary vagal affarents in the monkey: A survey of receptor activity. Basic Research in Cardiology, 72(4), 392–401. 409390 10.1007/BF02023598

